# Comparisons of short-term and long-term results between laparoscopic between open pancreaticoduodenectomy for pancreatic tumors: A systematic review and meta-analysis

**DOI:** 10.3389/fgene.2022.1072229

**Published:** 2023-01-20

**Authors:** Hongquan Qiu, Liang Zhang, Dongzhi Wang, Haiyan Miao, Yu Zhang

**Affiliations:** ^1^ Department of Surgery, Liuqiao Central Hospital, Nantong, China; ^2^ Department of General Surgery, Tengzhou Central People’s Hospital, Tengzhou, China; ^3^ Department of Hepatobiliary and Pancreatic Surgery, Affiliated Hospital of Nantong University, Nantong, China; ^4^ Department of General Surgery, The Sixth People’s Hospital of Nantong, Nantong, China; ^5^ Department of Laboratory Medicine, Haimen Hospital Affiliated to Xinglin College of Nantong University, Nantong, China

**Keywords:** laparoscopic, pancreaticoduodenectomy, pancreatic tumors, meta-analysis, surgery

## Abstract

**Objective:** The efficacy of pancreaticoduodenectomy and open pancreaticoduodenectomy for pancreatic tumors is controversial. The study aims to compare the efficacy of laparoscopic pancreaticoduodenectomy (LPD) and open pancreaticoduodenectomy (OPD) in the treatment of pancreatic tumors through systematic evaluation and meta-analysis.

**Methods:** PubMed, Embase, Cochrane Library and Web of science databases were searched for clinical studies on the treatment of pancreatic tumors with LPD and OPD. The end time for the searches was 20 July 2022. Rigorous inclusion and exclusion criteria were used to screen the articles, the Cochrane manual was used to evaluate the quality of the included articles, and the stata15.0 software was used for statistical analysis of the indicators.

**Results:** In total, 16 articles were included, including two randomized controlled trials and 14 retrospective studies. Involving a total of 4416 patients, 1275 patients were included in the LPD group and 3141 patients in the OPD group. The results of the meta-analysis showed that: the operation time of LPD was longer than that of OPD [WMD = 56.14,95% CI (38.39,73.89), *p* = 0.001]; the amount of intraoperative blood loss of LPD was less than that of OPD [WMD = −120.82,95% CI (−169.33, −72.30), *p* = 0.001]. No significant difference was observed between LPD and OPD regarding hospitalization time [WMD = −0.5,95% CI (−1.35, 0.35), *p* = 0.250]. No significant difference was observed regarding postoperative complications [RR = 0.96,95% CI (0.86,1.07, *p* = 0.463]. And there was no significant difference regarding 1-year OS and 3-year OS: 1-year OS [RR = 1.02,95% CI (0.97,1.08), *p* = 0.417], 3-year OS [RR = 1.10 95% CI (0.75, 1.62), *p* = 0.614%].

**Conclusion:** In comparison with OPD, LPD leads to less blood loss but longer operation time, therefore the bleeding rate per unit time of LPD is less than that of OPD. LPD has obvious advantages. With the increase of clinical application of LPD, the usage of LPD in patients with pancreatic cancer has very good prospect. Due to the limitations of this paper, in future studies, more attention should be paid to high-quality, multi-center, randomized controlled studies.

## 1 Introduction

Pancreatic cancer is the 12th most common malignant tumor in the world, and the seventh leading cause of death from cancer, with a 5-year survival rate of only 10% (Chang et al., 2022). In the past 25 years, the global burden of pancreatic cancer has doubled, and it is now ranking among the top 10 cancer deaths in more than 130 countries (Klein, 2021). According to the latest data of the American *Cancer* Association, there were about 60,430 new patients with pancreatic cancer in 2021, of which 48,220 died. It is expected that it will become the second leading cause of death from cancer in the United States within the next 20–30 years (Siegel et al., 2021). Among the European Union, pancreatic cancer is expected to surpass breast cancer and become the third leading cause of death related to cancer (Carioli et al., 2021). In China, the 5-year survival rate of pancreatic cancer has not been significantly ameliorated in the past 10 years, with a number of only 9.9%. With population growth, the growth of the aging propulation and the influence of Western lifestyles, the incidence rate of pancreatic cancer is expected to continue to rise in the next few years (Sun et al., 2020). As a digestive tract tumor with extremely poor prognosis, the main treatment for pancreatic cancer is still a multidisciplinary approach based on traditional open surgery (Neoptolemos et al., 2018; Chang et al., 2022). Pancreatoduodenectomy is the conventional surgical treatment for periampullary malignant tumors (Kawai and Yamaue, 2011). However, due to the limitations of OPD, such as the strong trauma that it causes, slow postoperative recovery, and long hospitalization time, as well as the continuously rising expectations for diagnosis and treatment methods from the patients, surgeons are constantly pursuing to make surgeries as minimally invasive as possible (Scialpi et al., 2016; Iovanna and Dusetti, 2017; Miller et al., 2020).

In recent years, surgical treatment for pancreatic cancer has gradually developed towards minimally invasive (Heestand et al., 2015). Minimally invasive surgery does not involve digestive tract anastomosis and reconstruction, with little technical difficulty and small incisions. The visual field can be magnified by 5–10 times under laparoscopy, which can enter into narrow spaces to obtain a field of vision unmatched by open surgery. The operative field is clearer, and the surgeries are more refined. Combined with advanced medical devices such as endoscopic cutter-staplers, ultrasonic knives, energy platforms, etc., it can very well achieve the dissection, dissociation, resection and anastomosis of some particular places, reduce the pain at the site of incision after surgery, contribute to the aesthetic appearance of the incision, and reduce trauma to the patients’ minds (Harrison et al., 2022). In addition, the magnified vision of laparoscopy provides more detailed anatomical opportunities for operators, and improves the safety of the surgery and the thoroughness of clearing (Pisters et al., 2001; Zhang et al., 2011; Cesaretti et al., 2017). However, the high recurrence rate after minimally invasive surgery, the high incidence of severe pancreatic fistula and the long operation time are still unsolved problems (Sciuto et al., 2014; Kuesters et al., 2018). Although the operation time of open surgery is short, the amount of blood loss during operation is large. Therefore, whether to choose open surgery or minimally invasive surgery for the treatment of pancreatic cancer is still a subject of great controversy (Zhang et al., 2011; Zhou et al., 2019). It is hoped that through this study, we can solve this controversy and provide a foundation for the selection of intervention (surgery) for the treatment of pancreatic cancer in clinic.

## 2 Methods

The study was registered with PROSPERO and followed PRISMA-P (the preferred reporting project for system review and meta-analysis scheme) guidelines.

### 2.1 Literature search

We searched the following English databases: PubMed, EMBASE, Cochrane Library and Web of science, with keywords such as laparoscopy, pancreatoduodenectomy, pancreatic cancer, etc. The search period was set from the foundation of the database to 20 July 2022. We searched the databases for clinical studies about LPD and OPD as treatment methods for pancreatic cancer. See Supplementary Table S1 for PubMed retrieval strategy.

### 2.2 Inclusion and exclusion criteria

Inclusion criteria: for those who met the diagnostic criteria for pancreatic cancer (Okusaka et al., 2020) and were older than 18 years old, LPD and OPD were used as intervention. The primary outcome indicators were: operation time, amount of intraoperative blood loss, hospitalization time, overall survival rate (OS). The secondary outcome indicators were: postoperative complications. Randomized controlled trials and retrospective studies were included in this study. Systematic reviews, repeatedly published articles, case reports, protocols, animal experiments, conference summaries, full texts unable to be obtained, articles without usable data; articles meeting the above criteria were excluded.

### 2.3 Literature and data extraction

Two researchers (HQQ and LZ) independently screened the studies to extract data. Preliminary screening was conducted by reading the titles and abstracts of the different literature, and for the ones that were easy to judge, literature screening was directly conducted; for literature that raised objections about whether they could be included, relevant teachers were consulted, and they were screened by directly downloading and reading the full texts. During the screening process, all the inclusion and exclusion criteria were strictly followed. Two researchers independently extracted the outcome indicators’ data from the studies, and the extracted information were cross checked in order to ensure the consistency of the extracted data. The extracted data included: first author, year of publication, experimental design, country, sample size, age, follow-up and outcome indicators.

### 2.4 Quality evaluation of the included literature

The quality evaluation of the included studies was independently completed by two researchers. For randomized controlled trials, the bias analysis evaluation tool provided by Cochrane Handbook for Systematic Reviews of Interventions 5.1.0 was used, in an effort to evaluate the quality of the included studies. The evaluation included seven aspects: generation of random sequence (selection bias), allocation concealment (selection bias), blinding of the implementers and participants (implementation bias), blinding of outcome evaluators (observation bias), integrity of data results (follow-up bias), selective reporting of research results (reporting bias), and other sources of bias. For retrospective studies, the Newcastle Ottawa Scale (NOS) was utilized to evaluate the quality of the cohort studies or case-control studies. The NOS scale includes two forms: one for cohort studies and one for case-control studies. The cohort study form includes eight items within three domains: study population selection, comparability between groups and result measurement. The case-control study form also includes eight items within three domains: study population selection, comparability between groups, and measurement of exposure factors. If the requirements are met, one point is scored, with a full score of nine points, and ≥five points is considered as high-quality literature.

### 2.5 Statistical analysis

Meta-analysis of data was carried out by using the Stata15.0 software. Continuous variables were expressed by weighted mean difference (WMD) and 95% confidence interval (CI), and binary variables were represented by relative risk (RR) and 95% confidence interval (CI). The heterogeneity of each study was tested. When *p* ≥ 0.1 and I2<50%, the heterogeneity was regarded as low, and the fixed effects model was used. When *p* < 0.1 and I2>50%, heterogeneity was considered to be present, therefore sensitivity analysis was used to investigate the source of heterogeneity. If the source of heterogeneity could not be determined, a random effects model was used to conduct meta-analysis for the literature. By observing whether the two sides of the funnel chart were symmetrical, it was judged whether or not the meta-analysis’ results contained publication bias. A value of *p* < 0.05 was considered as significant difference.

## 3 Results

### 3.1 Literature screening results

**FIGURE 1:**
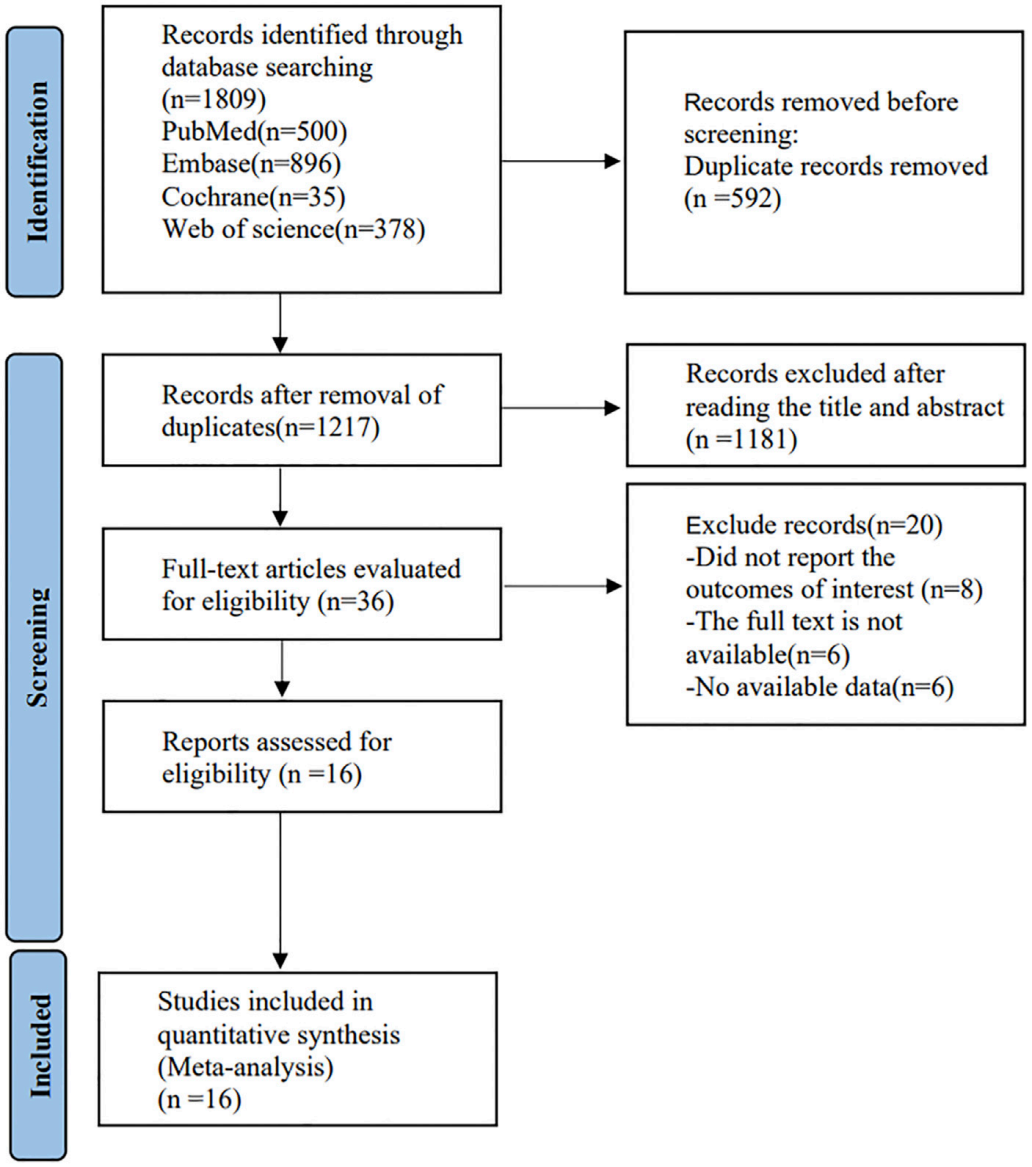
Flow chart of literature retrieval.

A total of 1809 articles were obtained through preliminary retrieval from the databases, 592 remained after removing duplicate articles, then 36 were obtained through preliminary screening by reading the titles and abstracts of the studies, and 16 (20–35) articles were finally included after reading the full texts. See Figure 1 for the flow diagram of literature screening.

### 3.2 Basic characteristics and quality evaluation of the included literature

Among the 16 included controlled studies about LPD and OPD for the treatment of pancreatic cancer, two were randomized controlled experiments (Palanivelu et al., 2017; Wang et al., 2021), and 14 were retrospective studies (Cho et al., 2009; Bao et al., 2014; Croome et al., 2014; Mendoza et al., 2015; Stauffer et al., 2017; Chapman et al., 2018; Kim et al., 2019; Tan et al., 2019; Choi et al., 2020; El Nakeeb et al., 2020; Han et al., 2020; Kwon et al., 2020; Weng et al., 2021; Zhang et al., 2022), involving a total of 4416 patients, including 1275 in the LPD group and 3141 in the OPD group. See Table 1 for literature characteristics, see Supplementary Table S2 and Supplementary Figures S1, S2 for the quality evaluation of the included studies.

**TABLE 1:** Characteristics of studies included.

Study	Study design	Country	Sample size (male)		Mean age (year)		Follow-up(M)	Outcome
			LPD	OPD	LPD	OPD		
Palanivelu et al (2017)	RCT	India	32 (18)	32 (22)	57.8	58.6	3	F1; F2; F3; F4; F5; F6; F7
Wang et al (2021)	RCT	China	297 (171)	297 (193)	61	60	3	F1; F2; F4; F5; F6; F8
Bao et al (2014)	Retrospective study	United States	28 (13)	28 (13)	68	67.7	3	F1; F2; F4; F5; F7; F8
Chapman et al (2018)	Retrospective study	United States	248 (132)	1520 (721)	79.6	79.5	60	F5; F7; F8; F9
Cho et al (2009)	Retrospective study	Japan	15 (6)	15 (7)	64	68	NA	F1; F2; F4; F5; F6
Choi et al (2020)	Retrospective study	Korea	27 (12)	34 (18)	63.35	63.35	60	F1; F2; F4; F5; F9; F10
Croome et al. (2014)	Retrospective study	United States	108 (51)	214 (131)	66.6	65.4	60	F1; F2; F4; F5; F9; F10
El Nakeeb et al. (2020)	Retrospective study	Egypt	37 (22)	74 (40)	NA	NA	NA	F1; F2; F4; F5; F6; F7
Han et al. (2020)	Retrospective study	Korea	104 (53)	113 (70)	61.5	64.5	3	F1; F2; F5; F6; F7; F8
Kim et al. (2019)	Retrospective study	Korea	58 (18)	91 (42)	49.5	56	60	F1; F2; F5; F6; F7; F9; F10
Kwon et al (2020)	Retrospective study	Korea	73 (41)	219 (114)	62.4	63.3	60	F6; F9; F10
Mendoza et al (2015)	Retrospective study	Korea	18 (10)	34 (21)	63.7	68.4	NA′	F1; F2; F4; F5; F8
Stauffer et al. (2017)	Retrospective study	United States	58 (32)	193 (96)	69.9	68.9	60	F1; F2; F5; F6; F9
Tan et al. (2019)	Retrospective study	Singapore	20 (11)	20 (11)	65	64	NA	F1; F2; F4; F5
Weng et al. (2021)	Retrospective study	China	105 (66)	210 (135)	64	62	60	F1; F2; F8; F9; F10
Zhang et al. (2022)	Retrospective study	China	47 (31)	47 (30)	57.64	57.57	50	F2; F4; F7; F9

F1: duration of operation; F2: blood loss; F3: Duration of ICU, stay; F4: blood transfusion; F5: duration of hospital stay; F6: postoperative complications; F7: readmission; F8: 90-Mortality; F9: overall survival; F10: disease-free survival.

### 3.3 Meta-analysis

#### 3.3.1 Operation time

**FIGURE 2:**
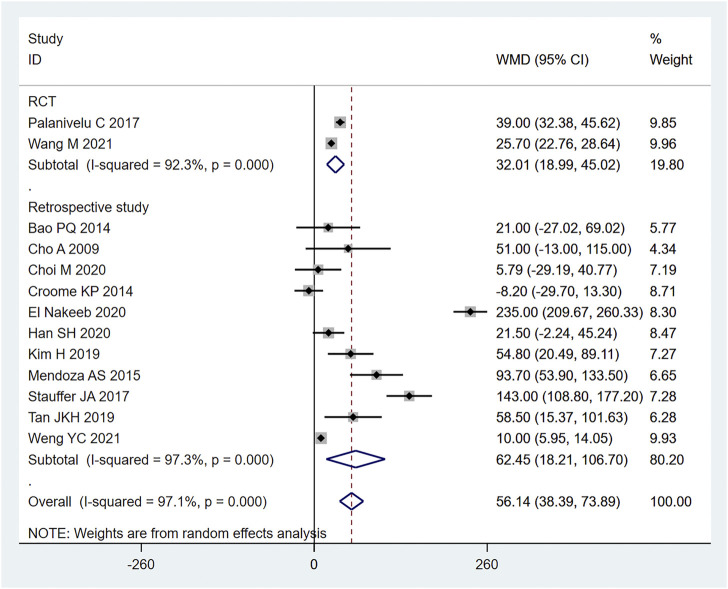
Forest plot of Meta-analysis of operation time.

**FIGURE 3:**
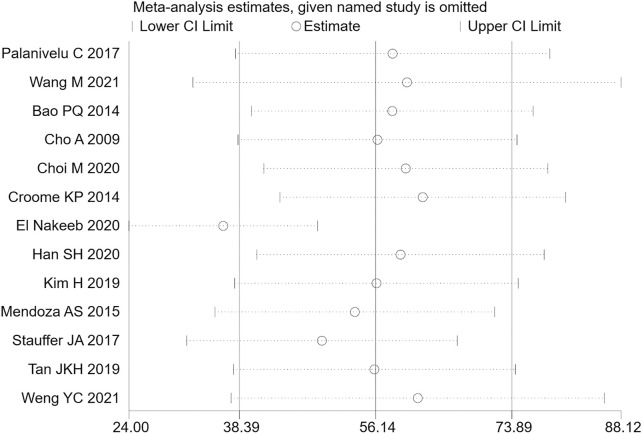
Sensitivity analysis of operation time.

Among the included studies, a total of 13 articles (Cho et al., 2009; Bao et al., 2014; Croome et al., 2014; Mendoza et al., 2015; Palanivelu et al., 2017; Stauffer et al., 2017; Kim et al., 2019; Tan et al., 2019; Choi et al., 2020; El Nakeeb et al., 2020; Han et al., 2020; Wang et al., 2021; Weng et al., 2021) mentioned the index of operation time. Among them, 907 cases were part of the LPD group and 1355 cases were in the OPD group. Therefore, the random effects model was used for meta-analysis. According to the heterogeneity test (I2 = 97.1%, *p* = 0.0001), the analysis results showed that the difference between the two groups was statistically significant [WMD = 56.14,95% CI (38.39,73.89), *p* = 0.001], indicating that the operation time of LPD was longer than that of OPD. As shown in Figure 2, subgroup analysis was then conducted according to literature type. The randomized controlled trial subgroup [WMD = 32.01,95% CI (18.99,45.02), *p* = 0.001], and the retrospective study subgroup [WMD = 62.45,95% CI (18.21106.70), *p* = 0.006]; the results showing that the operation time of LPD was longer than that of OPD in both randomized controlled studies and retrospective studies. The sensitivity analysis of this indicator was carried out after removing literature one by one, as shown in Figure 3.

#### 3.3.2 Intraoperative blood loss

**FIGURE 4:**
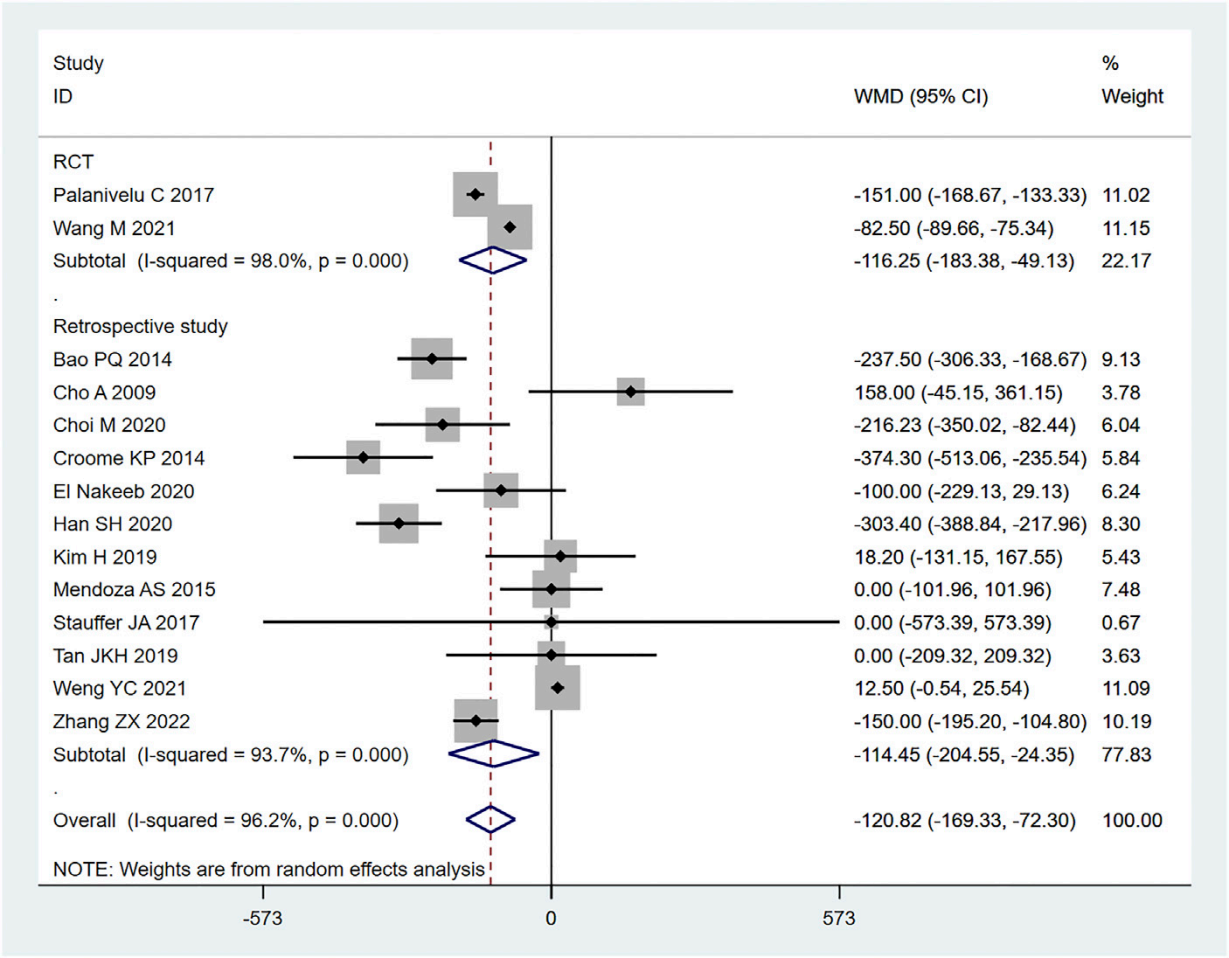
Forest plot of Meta-analysis of intraoperative blood loss.

**FIGURE 5:**
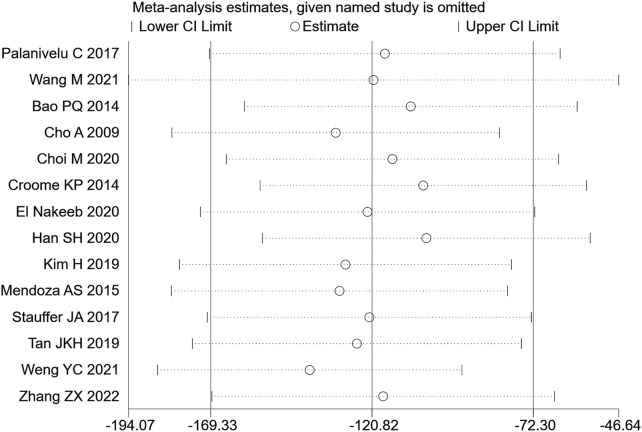
Sensitivity analysis of intraoperative blood loss.

14 (20, 22–28, 30, 31, 36) articles mentioned intraoperative blood loss as an indicator, including 954 cases in the LPD group and 1402 cases in the OPD group. The random effect model was used for meta-analysis. According to the heterogeneity test (I2 = 96.2%, *p* = 0.0001), the analysis results showed that the difference between the two groups was statistically significant [WMD = −120.82, 95% CI (−169.33, −72.30), *p* = 0.001], indicating that the amount of intraoperative blood loss in LPD was less than OPD. As shown in Figure 4, subgroup analysis was then conducted according to literature type. The randomized controlled trial subgroup [WMD = −116.25,95% CI (−183.38, −49.13), *p* = 0.013], and the retrospective study subgroup [WMD = −1114.45,95% CI (−204.55, −24.35), *p* = 0.006]; the results showing that the blood loss during LPD operation was less than that of OPD, in both randomized controlled studies and retrospective studies. Sensitivity analysis was conducted for this indicator by removing literature one by one, as shown in Figure 5.

#### 3.3.3 Hospitalization time

**FIGURE 6:**
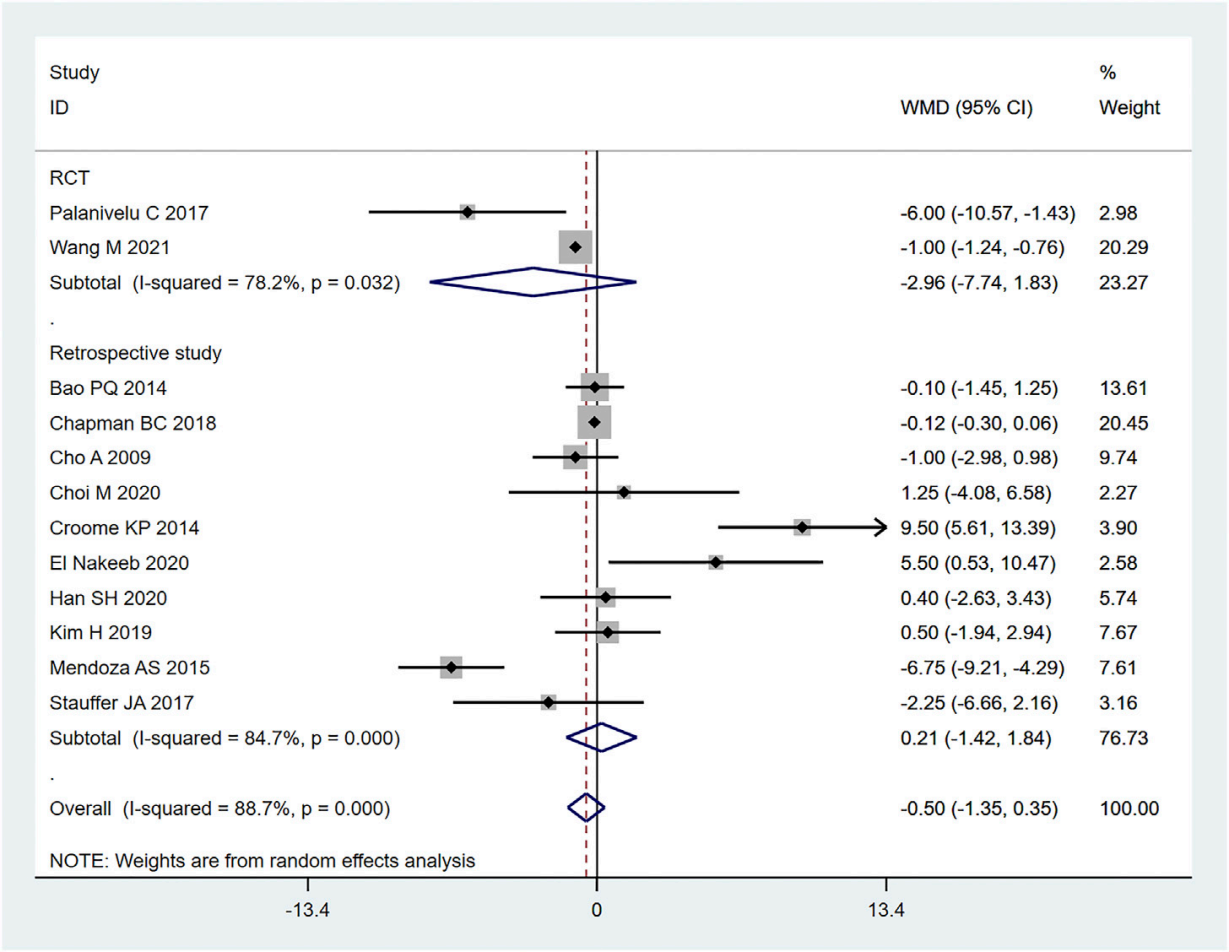
Forest plot of Meta-analysis of hospitalization time.

**FIGURE 7:**
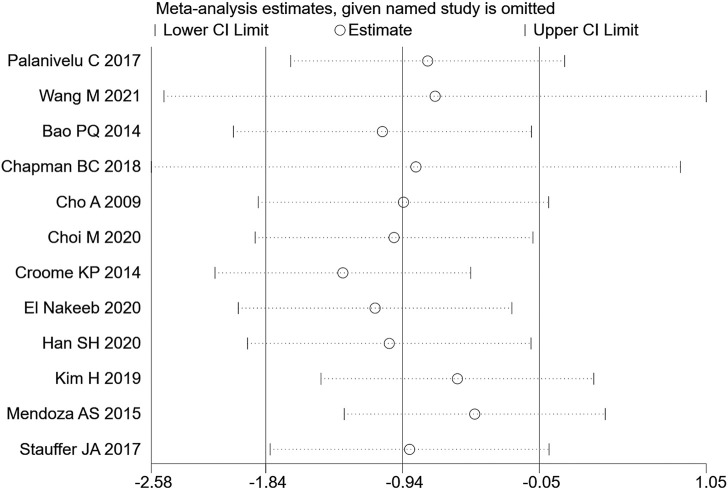
Sensitivity analysis of hospitalization time.

12 (20–23, 26, 27, 29, 30, 32–35) articles mentioned hospitalization time (length of stay in hospital), including 1030 cases from the LPD group and 2045 cases in the OPD group. The random effects model was used for meta-analysis. According to the heterogeneity test (I2 = 90.1%, *p* = 0.0001), the analysis results showed that the difference between the two groups was not statistically significant [WMD = −0.5,95% CI (−1.35,0.35), *p* = 0.250], indicating that there was no significant difference regarding the length of hospitalization between LPD and OPD. Subgroup analysis was then conducted according to literature type, as shown in Figure 6. The randomized controlled trial subgroup [WMD = −2.96,95% CI (−7.74,1.83), *p* = 0.226], and the retrospective study subgroup [WMD = 0.21,95% CI (−1.42,1.84), *p* = 0.801]; the results showing that, whether in randomized controlled trials or retrospective studies, there was no difference in hospilization time between LPD and OPD. Sensitivity analysis of this indicator was carried out by removing literature one by one, as shown in Figure 7.

#### 3.3.4 Postoperative complications

**FIGURE 8:**
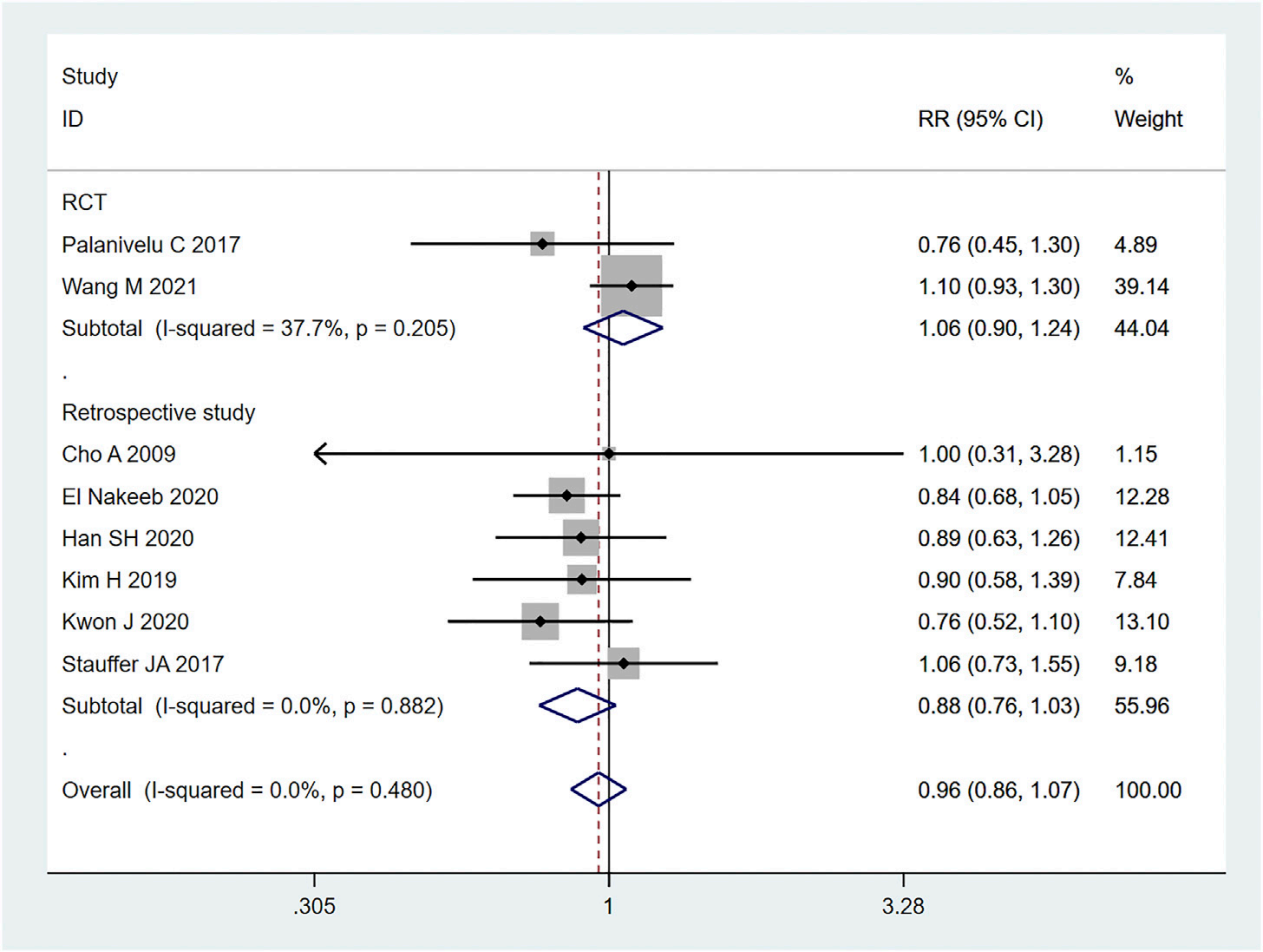
Forest plot of Meta-analysis of postoperative complications.

A total of 8 (23–28, 32, 35) studies mentioned postoperative complications, including 674 cases in the LPD group and 1034 cases in the OPD group. Therefore, meta-analysis was carried out on the fixed effects model. According to the heterogeneity test (I2 = 0%, *p* = 0.480), the analysis results showed that the difference between the two groups was not statistically significant [RR = 0.96,95% CI (0.86,1.07), *p* = 0.463], indicating that there no significant difference was observed between LPD and OPD regarding postoperative complications. As shown in Figure 8, subgroup analysis was carried out according to literature type. The randomized controlled trial subgroup [RR = 1.06,95% CI (0.90,1.24), *p* = 0.483], and the retrospective study subgroup [RR = 0.88,95% CI (0.76,1.03), *p* = 0.108]; the results showing that there was no difference regarding postoperative complications between LPD and OPD, whether in randomized controlled studies or retrospective studies.

#### 3.3.5 OS (overall survival)

**FIGURE 9:**
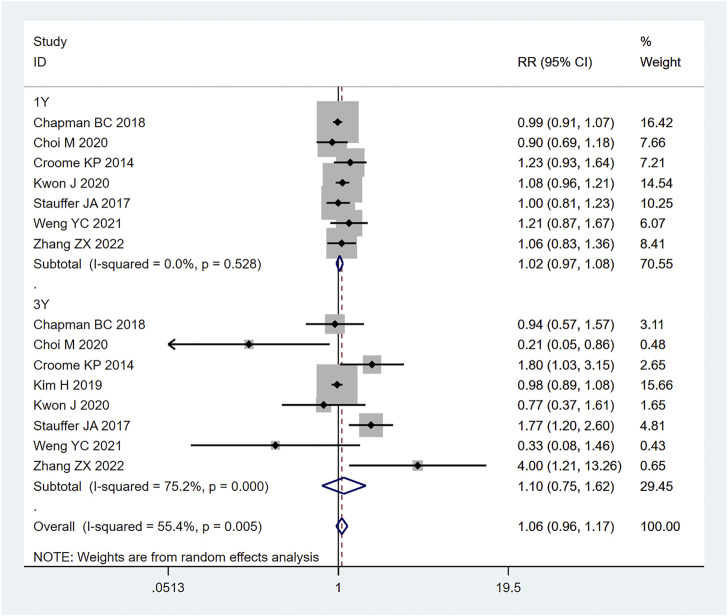
Forest plot of Meta-analysis of OS.

**FIGURE 10:**
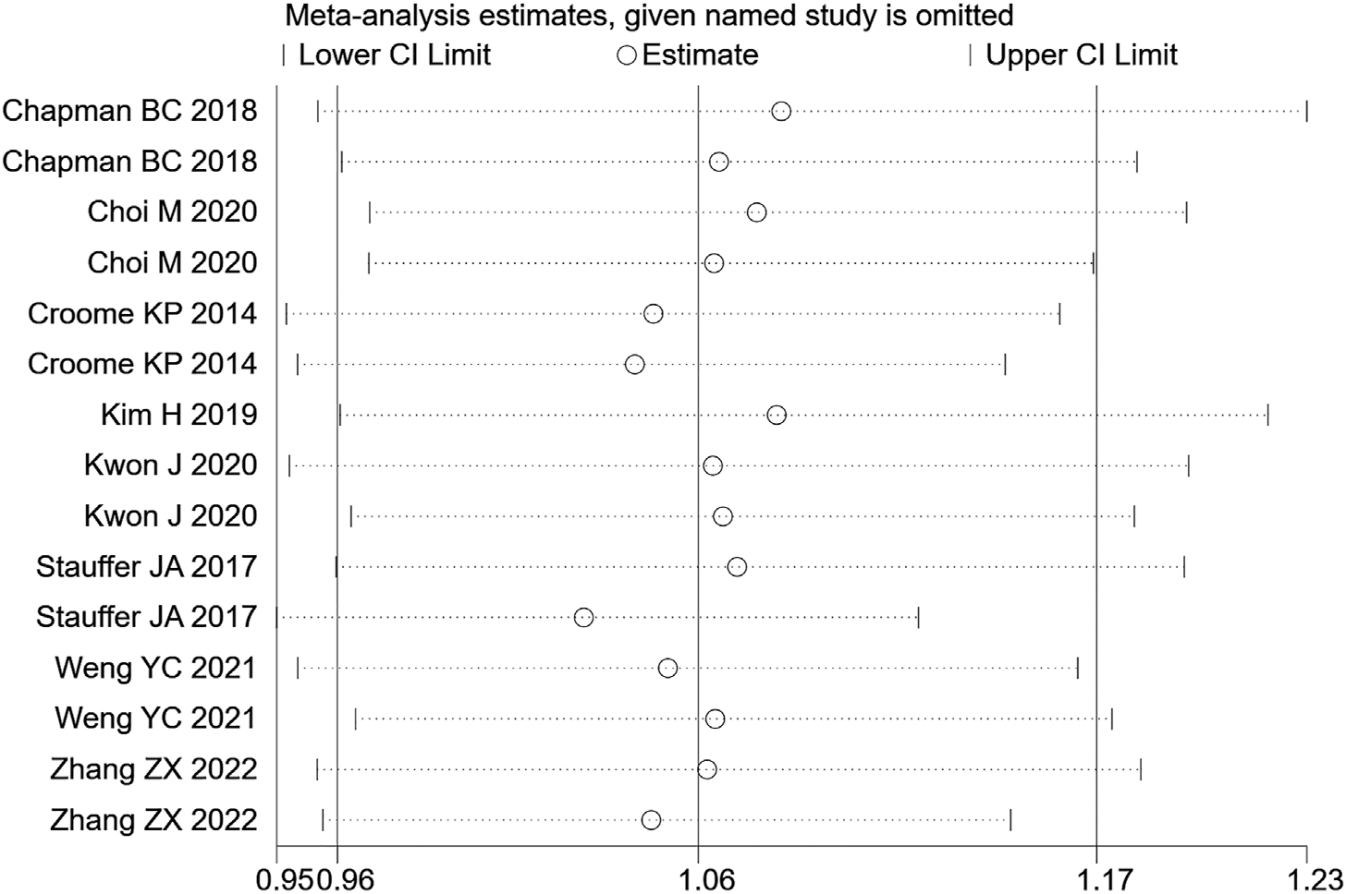
Sensitivity analysis of OS.

A total of 8 (20–22, 24, 26, 27, 30, 34) retrospective studies mentioned OS, respectively recording their 1-year OS and 3-year OS. Therefore, the random effects model was used for meta-analysis. According to the heterogeneity test (I2 = 55.4%, *p* = 0.005), the analysis results showed that the difference between the two groups was not statistically significant [RR = 1.96,95% CI (0.96,1.17), *p* = 0.216]. In the 1-year OS subgroup [RR = 1.02,95% CI (0.97,1.08), *p* = 0.417], and in the 3-year OS subgroup [RR = 1.10,95% CI (0.75, 1.62), *p* = 0.614]; whether in the 1-year OS or 3-year OS, no significant difference was observed between LPD and OPD, as shown in Figure 9. Sensitivity analysis was conducted for this indicator by removing the literature one by one, as shown in Figure 10.

### 3.4 Publication bias

The egger test was used to evaluate the publication deviation of the operative time and intraoperative blood loss in the article, and it was found that the operative blood loss *p* = 0.475 and the operative time egger test *p* = 0.147, which suggested that there was no publication bias in these two indexes. See Supplementary Figures S3, S4.

## 4 Discussion

Surgery is gradually heading towards a minimally invasive era, and the advantages of laparoscopic surgery are becoming increasingly prominent (Sammut et al., 2017). Before that, LPD has already been widely used to treat benign and low-grade malignant tumors located at the body and tail of the pancreas. Croome et al. (Croome et al., 2014) retrospectively analyzed clinical data of pancreatic cancer patients who underwent laparoscopic duodenectomy and open resection at Mayo Clinic from January 2008 to July 2013. They found that patients who underwent minimally invasive surgery recovered quickly after surgery, could receive adjuvant treatment sooner, and had their disease-free survival time after surgery extended.

Pancreatoduodenectomy involves many vessels and organs, with complex spatial structure. Laparoscopic technic can enlarge the visual field during operation, only causes little trauma, and results in fast postoperative recovery (Jiang et al., 2019). In this study, the amount of blood loss of pancreatic cancer patients during LPD was remarkably less than that of OPD [WMD = −120.82,95% CI (−169.33, −72.30), *p* = 0.001], but the operation time of LPD was longer than that of OPD [WMD = 56.14,95% CI (38.39,73.89), *p* = 0.001]. We believe that with an accumulation of operation volume, after passing through the learning curve of the challenge period of the third stage, the operation time of LPD can be significantly shortened, and will get closer to, or even less than that of OPD. Moreover, the bleeding volume of LPD is far less than that of OPD, so the bleeding rate of LPD per unit time should be lower than that of OPD. Therefore, it can be considered that LPD has obvious advantages over OPD. Nagakawa et al. (Nagakawa et al., 2021) conducted a cohort study on 42 patients (21 patients in the left superior mesenteric artery group and 21 patients in the right superior mesenteric artery group), and pointed out that jejunal vein and lower pancreaticoduodenal artery hemorrhage often occurred on the left side of the mesenteric artery. Nussbaum D et al. (Nussbaum et al., 2014) pointed out that a lesser amount of blood loss is related to lower complication rate; reducing pancreaticoduodenal bleeding during operation can reduce the incidence of serious postoperative complications. During LPD, it is easier to find important tissues such as blood vessels and nerves, due to a clearer fenestration and higher fineness, thus reducing the quantity of intraoperative blood loss. In addition, the amount of intraoperative blood loss is related to the surgical proficiency of the operator. Some studies have pointed out that the amount of intraoperative blood loss can be decreased with the increase of learning time. Overly long operation time, an increase of blood loss during operation, and occurrence of postoperative complications will all prolong the patient’s hospitalization time (Yeo et al., 1993). Therefore, the requirements for doctors’ surgical skills are also very high. The amount of time it takes to operate is closely related to the learning curve. Some scholars have divided the learning curve of LPD into three periods: 1–11 cases is considered as the initial learning period, 12–38 cases the period of technical competency, and 39–57 cases the challenge period (Godhi et al., 2017). At the initial stage, the operation time of LPD can be long. First, because the degree of freedom and agility that endoscopic surgery allows is not as good as that of laparotomy, and second, because of the complexity and difficulty of LPD itself. For LPD beginners, when dealing with the operation difficulties, such as separation and resection of the pancreatic uncinate process, clearing of deep abdominal lymph nodes, reconstruction of digestive tract (especially pancreaticoduodenal anastomosis), it will inevitably consume a lot of the surgeon’s time (Gerstenhaber et al., 2013; Meng et al., 2022), which further explains the results we obtained this time. For long-term outcomes, our study obtained: 1-year OS [RR = 1.02, 95% CI (0.97, 1.08), *p* = 0.417] and 3-year OS [RR = 1.10,95% CI (0.75, 1.62), *p* = 0.614], for which no significant difference was observed. It may be that the number of included studies was small, and the included articles did not fully and clearly record the factors that may affect the prognosis of patients, such as the learning curve of the surgeon, the degree of tumor differentiation after surgery, the tumor stage, or the integrity of clinical data (such as whether or not the patient received adjuvant therapy). In short, even if it is impossible to assume that patients of the LDP group had a longer survival time, the current studies show that LDP has no weaker oncological outcome than ODP regarding the treatment of pancreatic cancer.

This study still contains some limitations. First of all, the included articles did not include retrospective studies, and the sample size of some studies was small, with inevitable presence of selection bias, which lead to the lower evidence quality of this research. Second, most studies were single center studies, leading to high heterogeneity between studies. The number of cases included in most studies was small, therefore it was impossible to analyze the incidence of various types of postoperative complications in detail. Third, the follow-up time after surgery was inconsistent, which may be the source of potential heterogeneity of the study.

## 5 Conclusion

In summary, LPD involves less blood loss and longer operation time when compared with OPD, therefore the bleeding rate per unit time of LPD is less than that of OPD. LPD has obvious advantages. With the increase of clinical application of LPD, the usage of LPD in patients with pancreatic cancer has a good prospect. Due to the limitations of this paper, in future studies, more attention should be paid to high-quality, multi-center, randomized controlled studies.

## Data Availability

The original contributions presented in the study are included in the article/Supplementary Material, further inquiries can be directed to the corresponding author.
