# Supplementary material for: Comparisons of short-term and long-term results between laparoscopic between open pancreaticoduodenectomy for pancreatic tumors: A systematic review and meta-analysis
Source: Front Genet. 2023 Jan 20;13:1072229. doi: 10.3389/fgene.2022.1072229 (PMC9894883; doi:10.3389/fgene.2022.1072229)
Supplement: Supplementary file 1 [file Table1.DOCX]

Supplementary Material

# Supplementary Figures


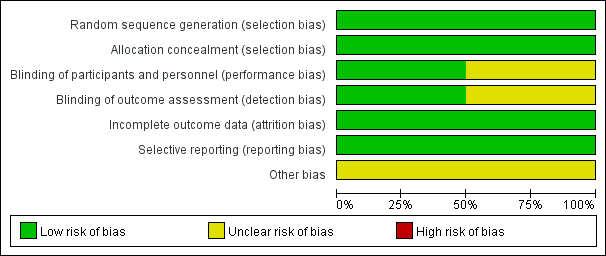


**Figure S1.** Risk of bias graph


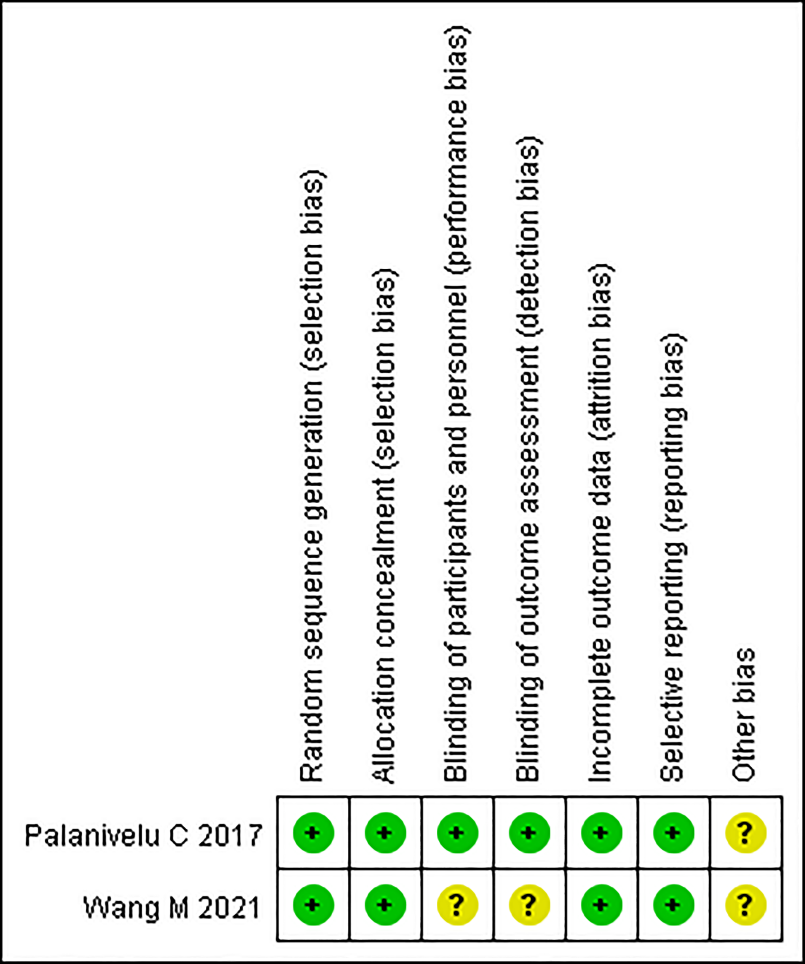


**Figure S2.** risk of bias summary


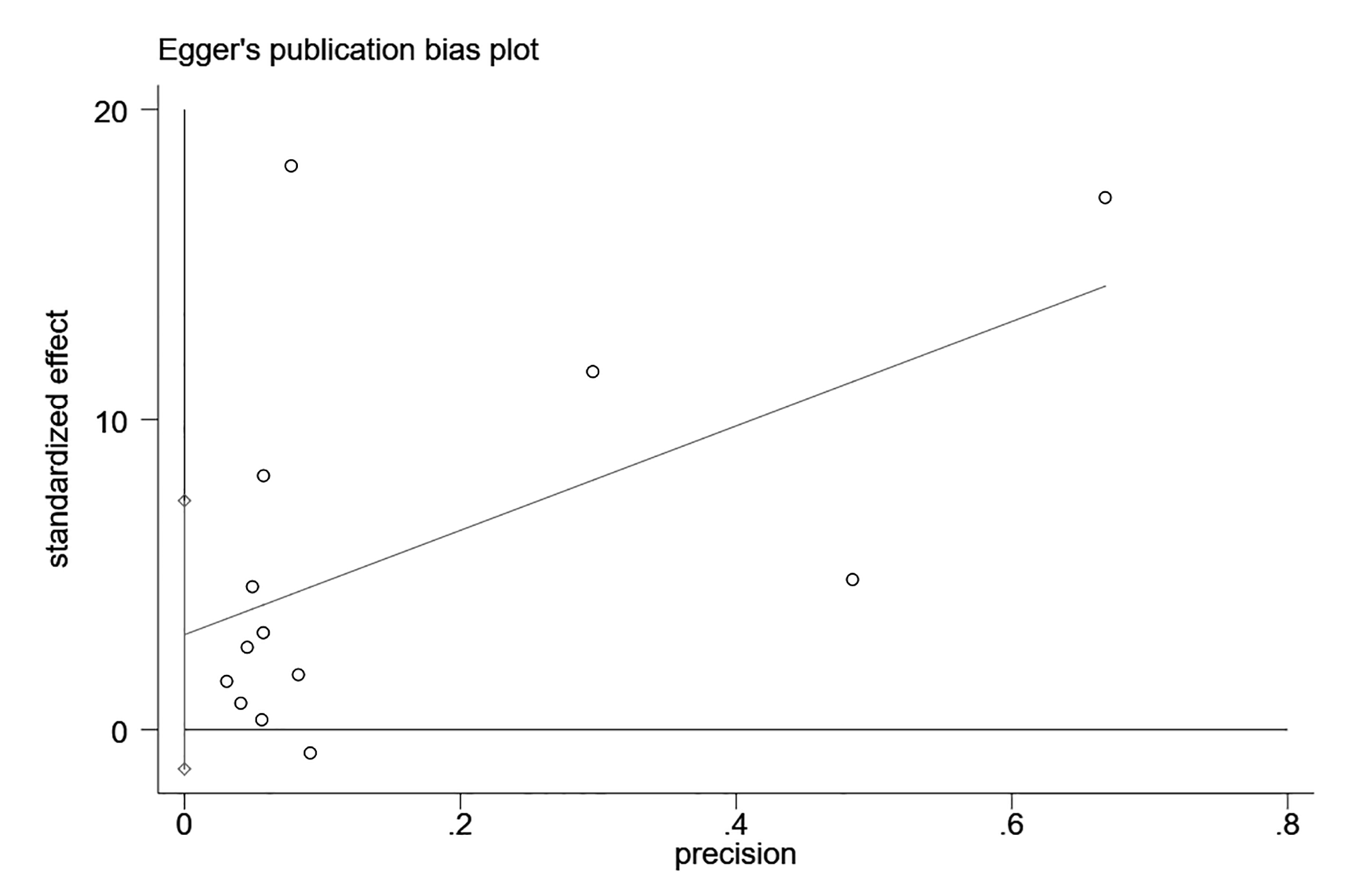


**Figure S3.** Egger’s test of operation time


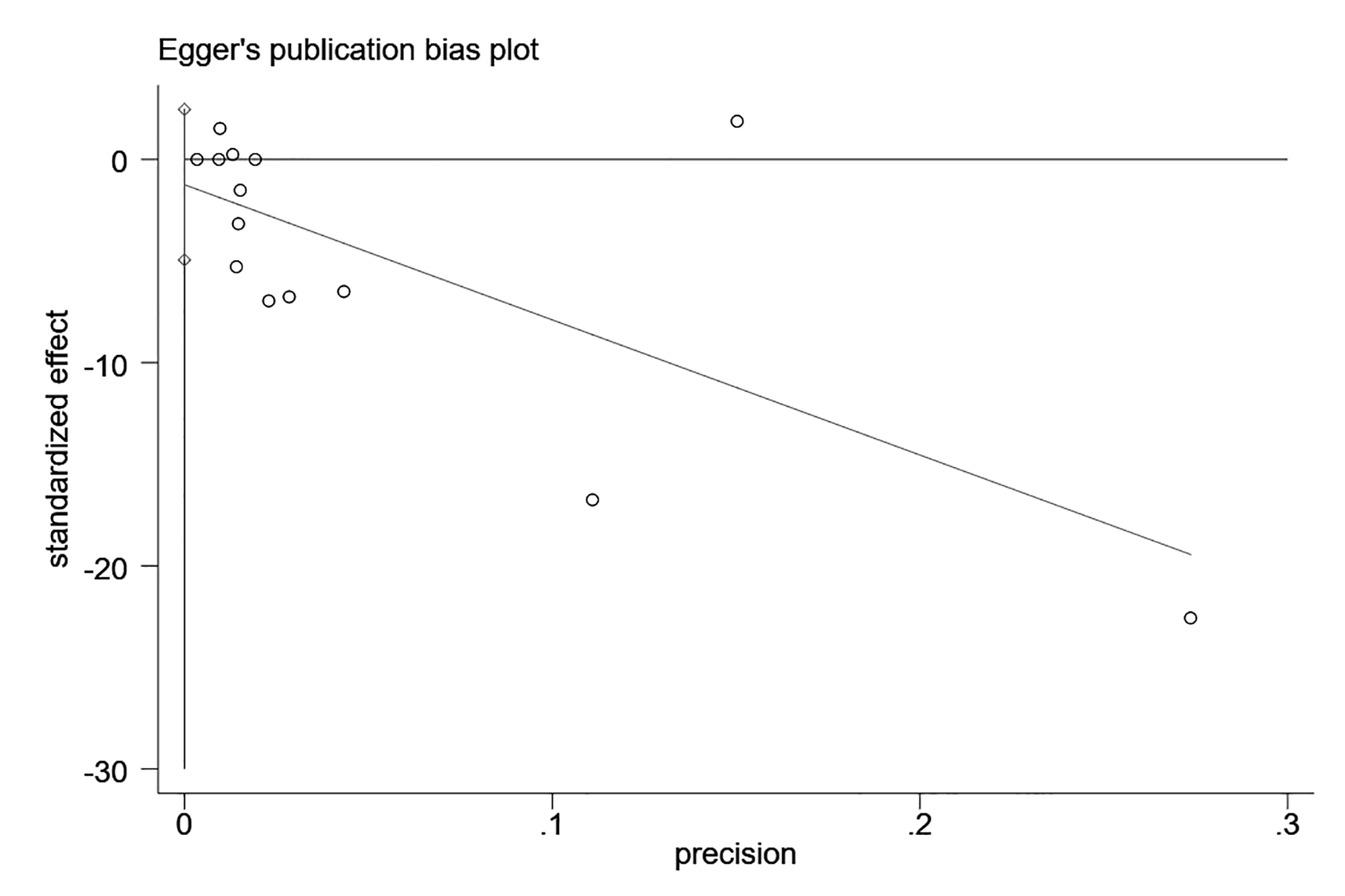


**Figure S4.** Egger’s test of blood loss during operation


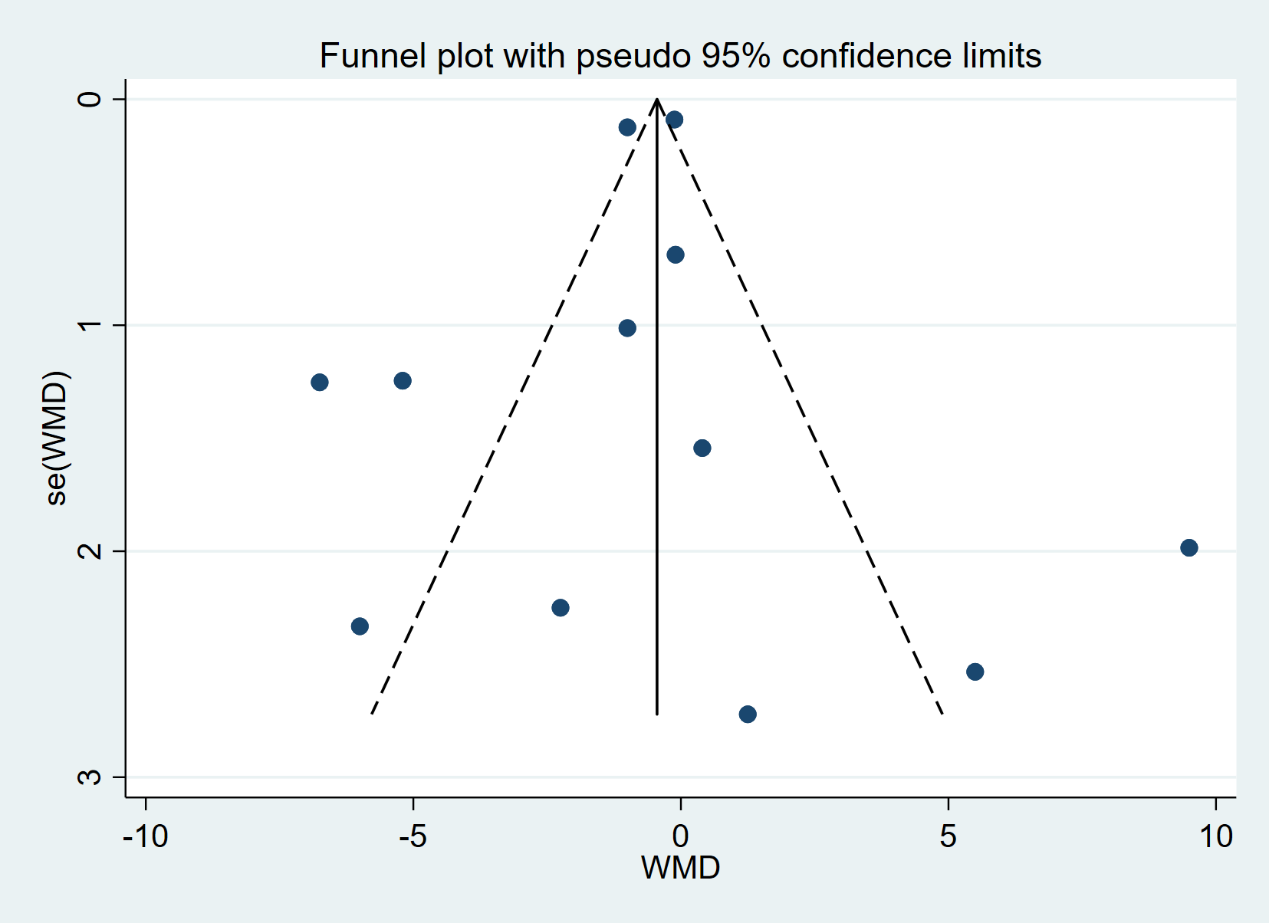


**Figure S5.** Funnel plot of hospitalization time


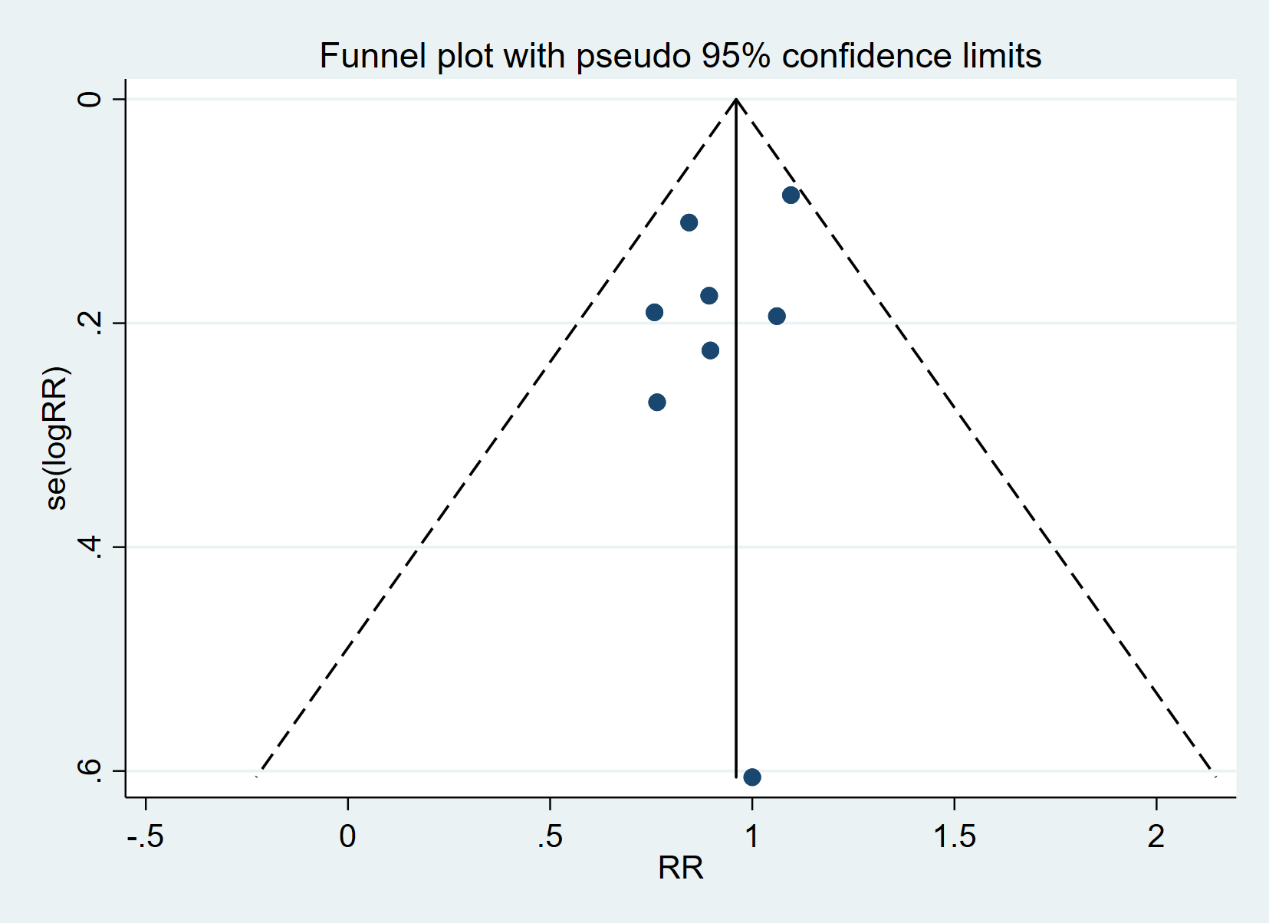


**Figure S6.** Funnel plot of postoperative complications


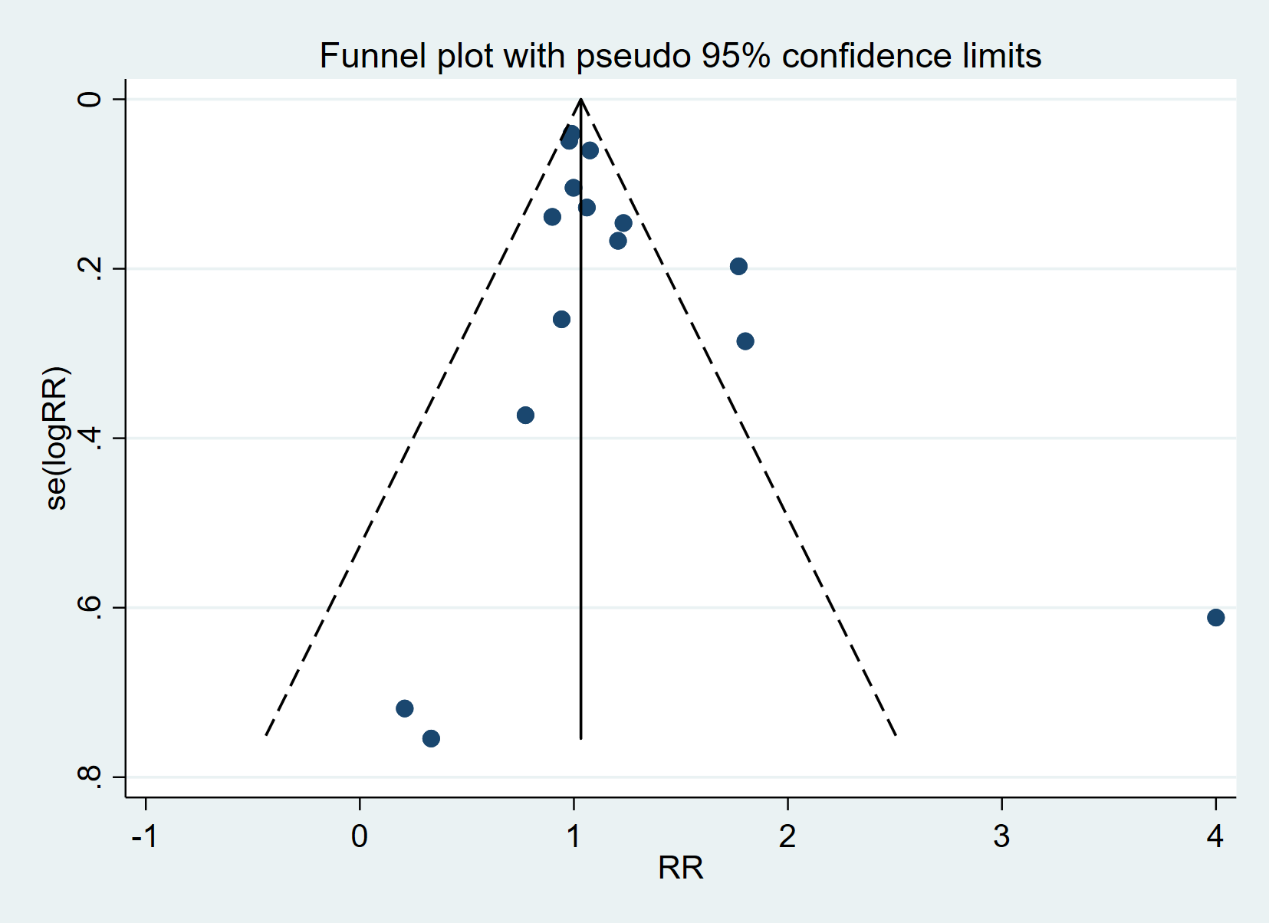


**Figure S7.** Funnel plot of OS

# Supplementary Tables

**Table S1.** PubMed retrieval strategy

| Search number | Query |
| --- | --- |
| 10 | #3 AND #6 AND #9 |
| 9 | #7 OR #8 |
| 8 | (((((((((((((((((Pancreatic Neoplasms[Title/Abstract]) OR (Neoplasm, Pancreatic[Title/Abstract])) OR (Pancreatic Neoplasm[Title/Abstract])) OR (Pancreas Neoplasms[Title/Abstract])) OR (Neoplasm, Pancreas[Title/Abstract])) OR (Neoplasms, Pancreas[Title/Abstract])) OR (Pancreas Neoplasm[Title/Abstract])) OR (Neoplasms, Pancreatic[Title/Abstract])) OR (Cancer of Pancreas[Title/Abstract])) OR (Pancreas Cancers[Title/Abstract])) OR (Pancreas Cancer[Title/Abstract])) OR (Cancer, Pancreas[Title/Abstract])) OR (Cancers, Pancreas[Title/Abstract])) OR (Pancreatic Cancer[Title/Abstract])) OR (Cancer, Pancreatic[Title/Abstract])) OR (Cancers, Pancreatic[Title/Abstract])) OR (Pancreatic Cancers[Title/Abstract])) OR (Cancer of the Pancreas[Title/Abstract]) |
| 7 | "Pancreatic Neoplasms"[Mesh] |
| 6 | #4 OR #5 |
| 5 | (((((Pancreaticoduodenectomies[Title/Abstract]) OR (Pancreaticoduodenectomy[Title/Abstract])) OR (Pancreatoduodenectomy[Title/Abstract])) OR (Pancreatoduodenectomies[Title/Abstract])) OR (Duodenopancreatectomy[Title/Abstract])) OR (Duodenopancreatectomies[Title/Abstract]) |
| 4 | "Pancreaticoduodenectomy"[Mesh] |
| 3 | #1 OR #2 |
| 2 | (((((((((((((((((((Laparoscopy[Title/Abstract]) OR (Laparoscopies[Title/Abstract])) OR (Celioscopy[Title/Abstract])) OR (Celioscopies[Title/Abstract])) OR (Peritoneoscopy[Title/Abstract])) OR (Peritoneoscopies[Title/Abstract])) OR (Surgical Procedures, Laparoscopic[Title/Abstract])) OR (Laparoscopic Surgical Procedure[Title/Abstract])) OR (Procedure, Laparoscopic Surgical[Title/Abstract])) OR (Procedures, Laparoscopic Surgical[Title/Abstract])) OR (Surgery, Laparoscopic[Title/Abstract])) OR (Laparoscopic Surgical Procedures[Title/Abstract])) OR (Laparoscopic Surgery[Title/Abstract])) OR (Laparoscopic Surgeries[Title/Abstract])) OR (Surgeries, Laparoscopic[Title/Abstract])) OR (Laparoscopic Assisted Surgery[Title/Abstract])) OR (Laparoscopic Assisted Surgeries[Title/Abstract])) OR (Surgeries, Laparoscopic Assisted[Title/Abstract])) OR (Surgery, Laparoscopic Assisted[Title/Abstract])) OR (Surgical Procedure, Laparoscopic[Title/Abstract]) |
| 1 | "Laparoscopy"[Mesh] |

## Table S2. NOS for Retrospective study

| Study | A | B | C | D | E | F | G | H | Total score |
| --- | --- | --- | --- | --- | --- | --- | --- | --- | --- |
| Bao PQ 2014 | 1 | 1 | 1 | 1 | 2 | 1 | 1 | 1 | 9 |
| Chapman BC 2018 | 1 | 1 | 1 | 1 | 2 | 1 | 1 | 1 | 9 |
| Cho A 2009 | 1 | 1 | 1 | 1 | 0 | 1 | 1 | 1 | 7 |
| Choi M 2020 | 1 | 1 | 1 | 1 | 2 | 1 | 1 | 1 | 9 |
| Croome KP 2014 | 1 | 1 | 1 | 1 | 2 | 0 | 1 | 1 | 8 |
| El Nakeeb 2020 | 0 | 1 | 1 | 1 | 0 | 1 | 1 | 1 | 6 |
| Han SH 2020 | 1 | 1 | 1 | 1 | 2 | 1 | 1 | 1 | 9 |
| Kim H 2019 | 1 | 1 | 1 | 1 | 2 | 0 | 1 | 1 | 7 |
| Kwon J 2020 | 1 | 1 | 1 | 1 | 1 | 1 | 1 | 1 | 8 |
| Mendoza AS 2015 | 1 | 1 | 1 | 1 | 1 | 1 | 1 | 1 | 8 |
| Stauffer JA 2017 | 1 | 0 | 1 | 1 | 2 | 1 | 1 | 1 | 8 |
| Tan JKH 2019 | 1 | 0 | 1 | 1 | 2 | 1 | 1 | 1 | 8 |
| Weng YC 2021 | 1 | 1 | 1 | 0 | 0 | 1 | 1 | 1 | 6 |
| Zhang ZX 2022 | 1 | 1 | 1 | 0 | 2 | 1 | 1 | 1 | 8 |

A definition of cases; B representativeness of cases; C. Selection of controls. D. Definition of comparison; E Case and control comparability based on design or analysis; F determination of exposure; G whether the same determination method was used for case and control exposures; H No response rate
